# Breakthrough in cardiac arrest: reports from the 4th Paris International Conference

**DOI:** 10.1186/s13613-015-0064-x

**Published:** 2015-09-17

**Authors:** Peter J. Kudenchuk, Claudio Sandroni, Hendrik R. Drinhaus, Bernd W. Böttiger, Alain Cariou, Kjetil Sunde, Martin Dworschak, Fabio Silvio Taccone, Nicolas Deye, Hans Friberg, Steven Laureys, Didier Ledoux, Mauro Oddo, Stéphane Legriel, Philippe Hantson, Jean-Luc Diehl, Pierre-Francois Laterre

**Affiliations:** University of Washington, Seattle, USA; Department of Anaesthesiology and Intensive Care, Catholic University School of Medicine, Rome, Italy; Department of Anaesthesiology and Intensive Care Medicine, University of Koeln, Cologne, Germany; Medical Intensive Care Unit, AP-HP, Cochin Hospital, Paris, France; Paris Descartes University and Sorbonne Paris Cité-Medical School and INSERM U970 (Team 4), Cardiovascular Research Center, European Georges Pompidou Hospital, Paris, France; Division of Emergencies and Critical Care, Department of Anaesthesiology, Surgical Intensive Care Unit Ullevål, Oslo University Hospital, Oslo, Norway; Division of Cardiothoracic and Vascular Anesthesia and Intensive Care Medicine, Vienna General Hospital, Medical University Vienna, Vienna, Austria; Department of Intensive Care, Laboratoire de Recherche Experimentale, Erasme Hospital, Brussels, Belgium; Medical Intensive Care Unit, AP-HP, Lariboisière University Hospital, Inserm U942, Paris, France; Anaesthesiology and Intensive Care Medicine, Skåne University Hospital, Lund University, Lund, Sweden; Coma Science Group, Cyclotron Research Centre, University of Liège and Liège 2 Department of Neurology, University Hospital of Liège, Liège, Belgium; Coma Science Group, Cyclotron Research Centre, University of Liège and Department of Intensive Care Medicine, University Hospital of Liège, Liège, Belgium; Department of Intensive Care Medicine, Faculty of Biology and Medicine, CHUV-University Hospital, Lausanne, Switzerland; Intensive Care Unit, Centre Hospitalier de Versailles, Le Chesnay, France; Department of Intensive Care, Cliniques Universitaires Saint-Luc, Université Catholique de Louvain, Brussels, Belgium; Medical Intensive Care Unit, AP-HP, European Georges Pompidou Hospital, Paris Descartes University and Sorbonne Paris Cité-Medical School, Paris, France; Department of Intensive Care, Cliniques Universitaires Saint-Luc, Université Catholique de Louvain Brussels, Brussels, Belgium

**Keywords:** Cardiac arrest, Cardio-pulmonary resuscitation, Targeted temperature management, Therapeutic hypothermia, Persistent vegetative state, Minimally conscious state, Organ donation

## Abstract

*Jean-Luc Diehl* The French Intensive Care Society organized on 5th and 6th June 2014 its 4th “Paris International Conference in Intensive Care”, whose principle is to bring together the best international experts on a hot topic in critical care medicine. The 2014 theme was “Breakthrough in cardiac arrest”, with many high-quality updates on epidemiology, public health data, pre-hospital and in-ICU cares. The present review includes short summaries of the major presentations, classified into six main chapters:

- Epidemiology of CA
- Pre-hospital management
- Post-resuscitation management: targeted temperature management
- Post-resuscitation management: optimizing organ perfusion and metabolic parameters
- Neurological assessment of brain damages
- Public healthcare

## Review

### Epidemiology of CA

#### Out-of-hospital cardiac arrest in 2014: incidence, outcome and disparities

##### Peter Kudenchuk

Out-hospital-cardiac arrest (OHCA) remains a common public health problem. Each year, cardiac arrest claims more than 424,000 lives in the United States, 300,000 lives in Europe, and upwards of 3.7 million lives worldwide. Resuscitation is typically conducted in accordance with a uniform algorithmic approach embodied in the “chain of survival” which emphasizes the importance of early activation of emergency medical services, prompt CPR, rapid defibrillation, advanced cardiac life support and post-cardiac arrest care. This, in fact might be said to be the only uniform aspect of cardiac arrest, which is otherwise riddled with disparities. The first of these disparities lies in how few patients who are successfully resuscitated from cardiac arrest ultimately survive to hospital discharge. In Seattle, approximately 60 % of patients in whom cardiac arrest presents as ventricular fibrillation (VF) are successfully resuscitated and admitted to hospital; yet only about half of these (30 %) typically survive to hospital discharge. When cardiac arrest presents as asystole or pulseless electrical activity (PEA), outcomes are strikingly worse: only 20–30 % of such patients are successfully resuscitated, and an even smaller proportion of these, as few as 2 in 10—ranging from 2 to 5 % of patients, survive to hospital discharge [1].

A second disparity lies in the changing incidence of rhythms that precipitate cardiac arrest. In Seattle during the decade of the 1970s, VF accounted for approximately 60 % of all out-of-hospital cardiac arrests treated by emergency medical services (EMS), whereas the remainder of acute rhythm presentations were equally divided between asystole and PEA. In the ensuing years, the proportion of cardiac arrests caused by VF has declined to 25–30 % of cases, such that now asystole and PEA represent the most common presenting rhythms. Extrapolating these incidence data from Seattle to the United States census, the annual rate of cardiac arrest due to ventricular fibrillation declined from about 85 persons per 100,000 in 1980 to 38/100,000 in 2000 [1]. Data from the Resuscitation Outcomes Consortium estimated a further decline in the incidence of VF cardiac arrest to 17.4 adults/100,000 in 2011 [2]. These statistics, coupled with the known worse survival prognosis of patients in whom cardiac presents as a non-shockable rhythm, pose a new and major challenge for the present and future treatment of this emerging “new wave” of cardiac arrest victims.

**Fig. 1:**
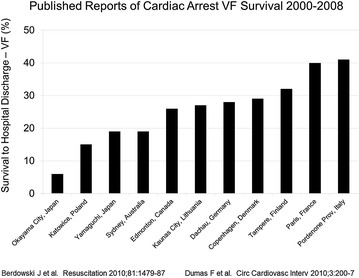
Survival to hospital discharge from out-of-hospital ventricular fibrillation in various communities outside the United States

A third disparity lies in the marked differences in outcome from cardiac arrest between communities across Europe and the United States. For cardiac arrest due to VF, survival can vary significantly between major cities by many-fold (Fig. 1) [3–5]. To some extent, such differences in outcome are explained by inaccuracies in record keeping. For example, few communities actually report their incidence of cardiac arrest and outcome [6]. And even among those that do, complete capture of all cases of cardiac arrest and the reliability of their survival data can be questionable, accounting for some of the variability in outcomes that are reported. This said, record keeping alone does not entirely explain these discrepancies. Identifying and targeting remediable causes of differences in survival between communities is imperative if we are to assure citizens that they are comparably “safe” from death by cardiac arrest in whatever locale they call home. Clinical factors that are known to account for differences in survival outcome from cardiac arrest include patient characteristics—such as age, gender, and co-morbidities; and by the circumstances of the arrest—such as whether witnessed by bystanders, the location of its occurrence, and by the presenting arrest rhythm. Most would regard these as “factors of fate” and as such not alterable. Conversely, the components of prehospital emergency medical care, including rapid dispatch, dispatcher-assisted CPR, EMS training and time-to-treatment, are all potentially correctable factors. Targeting these aspects of prehospital care affords an opportunity to change outcome for the better after cardiac arrest. Among these, perhaps the one that can be most readily and immediately implemented is high-performance CPR.

**Fig. 2:**
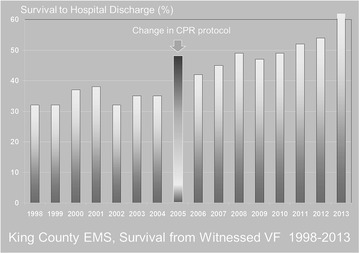
The impact of instituting high-performance CPR in King County, Washington, in 2005 on survival from witnessed out-of-hospital cardiac arrest due to ventricular fibrillation

High-performance CPR consists of training with meticulous attention to the details of performing CPR to the best known prescribed parameters of chest compression rate, depth, full chest recoil, and minimized interruptions, and applying strict compliance standards (e.g. a compression fraction of no less than 85 %) for their performance during resuscitation. In addition to striving for “letter perfect” CPR performance, it also involves a system of accountability, whereby feed-back of CPR performance derived from a review of recordings of field resuscitations is consistently conveyed back to providers for further possible improvement. Deploying such a high-performance CPR protocol in King County, Washington, starting in 2005 resulted in a significant improvement in survival from both cardiac arrest due to ventricular fibrillation (Fig. 2) [7], as well as asystole/PEA [8] that has been sustained in the ensuing years. The attractiveness of high-performance CPR is that it is relatively inexpensive to deploy (can be easily added to existing EMS training programs), does not require special equipment (hands only), and has demonstrated that it can improve outcome for virtually all presentations of cardiac arrest.

While many of the identified disparities in cardiac arrest will continue to pose challenges for its management, a focus on improving systems-of-care, particularly deployment of high-performance letter-perfect CPR, offers the promise of a practical intervention that can be implemented immediately and an effort that promises to improve survival outcomes in any community.

#### Effectiveness of rapid response systems for prevention of cardiac arrest

##### Claudio Sandroni

**Fig. 3:**
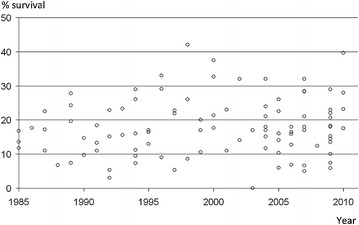
The 25-year trend of rates of survival to discharge after resuscitation from in-hospital cardiac arrest in 100 observational studies, 1985–2010

Despite the immediate availability of qualified life support, the outcome of in-hospital cardiac arrest (IHCA) remains poor. Survival to discharge after IHCA rarely exceeds 20 % [9] and it has remained stable in the last 25 years (Fig. 3). Rapid response systems (RRS) have been established to prevent IHCA in non-critical care areas of the hospital [10]. Those systems are based on timely detection of deteriorating patients by the ward personnel (the afferent limb of the system), who will therefore summon a medical emergency team (MET; the efferent limb of the system), whose roles are to stabilize the patient in the ward or escalate the level of care. Although the theory underlying RRS is compelling, there is no definite evidence that their implementation improves patient outcome. The major problem in evaluating the effectiveness of RRS is the choice of the outcome measure.

The first endpoint for a study addressing the effectiveness of RRS could be the rates of unexpected cardiac arrests occurring outside intensive care units (ICUs), that is, the rates of cardiac arrest occurring in ward patients for whom there is no do-not-attempt-resuscitation (DNAR) order. This endpoint, however, is potentially biased by the fact that one of the tasks of METs is to identify ward patients for whom a resuscitation would be inappropriate. Therefore, part of the observed reduction in the rate of unexpected cardiac arrest after the implementation of a RRS is because the fraction of expected cardiac arrests is increased by placement of a DNAR order.

Another endpoint for measuring RRS effectiveness is the reduction of unplanned ICU admissions. The rationale is that the introduction of RRS should increase the number of ICU admissions that are planned early, before further deterioration occurs, and decrease those occurring as emergency admissions after resuscitation from cardiac arrest. This model has been indirectly demonstrated for ICU admissions from Emergency Department [11] where an earlier transfer to ICU has been demonstrated to decrease both ICU and hospital mortality. However, this is not always the case with RRS. In one large American before-and-after study [12] in which almost half of the MET interventions resulted in an ICU admission, the implementation of the RRS was followed by a reduction of non-ICU codes but it did not translate in a reduction of hospital mortality. In that study, mortality in patient transferred from ward to ICU was very high, and problems of patient selection, appropriateness and timeliness of ICU transfer have been advocated to explain these results.

The third, and most comprehensive endpoint for RRS effectiveness is hospital mortality. Unfortunately, meta-analyses of available evidence on this endpoint showed conflicting results, with some studies showing benefit and others showing no or only non-significant reduction of hospital mortality after RRS implementation [13]. Moreover, the quality of evidence is relatively low, with almost all studies having a before-and-after design, which make them prone to bias due to secular trends unrelated to the study intervention or to changes in hospital case mix, a variable which is difficult to adjust for.

The ultimate strategy for assessing RSS effectiveness would be a randomized trial with concurrent cohorts, which would allow the investigators to control for most possible confounders. However, this solution is hampered by both ethical and implementation issues. Randomization at individual patient level for interventions which are commonly believed to be beneficial would in fact be ethically questionable. Cluster randomization is ethically acceptable, but difficult to implement because RRS intervention cannot be blinded, and contamination between the two study arms would be unavoidable, as did actually occur in the MERIT trial, the only randomized study conducted since now on RRS [14]. Another major implementation issue in that trial, as in general for RRS, was an afferent limb failure [15], due to an incomplete compliance of the ward personnel with the MET calling criteria.

A final issue is reproducibility. The vast majority of studies have been made in UK or Australian–New Zealand systems, a minority of studies have been conducted in US and only very few studies have been conducted in other World areas as Continental Europe or Asia. The effectiveness of an RRS depends on the nature and the quantity of the urgent, unmet patients’ needs in general wards. This model may not work in places where the severity of patients in general wards, the education of the ward personnel or the resource availability is different from that of places where this model was developed.

In summary, there are different ways of measuring the effectiveness of RRSs. The major include the rate of unexpected CA outside ICU, the rate of unplanned ICU admissions, and hospital mortality. All these outcome measures have limitations and are prone to bias. The level of evidence supporting the effectiveness of RRSs is relatively low and almost all studies have a before-and-after design. Despite the ethical and implementation difficulties, high-quality randomized trials are warranted to reliably assess the effectiveness of RRS.

### Pre-hospital management

#### CPR quality has a deep impact

##### Hendrik Drinhaus and Bernd Böttiger

Despite enormous efforts in recent years to improve quality of cardiopulmonary resuscitation (CPR) by the development of new CPR-guidelines and to enhance post-resuscitation care particularly by the introduction of mild therapeutic hypothermia and early coronary intervention, survival rates after OHCA remain unsatisfyingly low in many countries. As a prerequisite for successful post-resuscitation care on the Intensive Care Unit, high-quality CPR must be started as early as possible. As advanced life support (ALS) by emergency medical services (EMS) can only be expected to be commenced several minutes after OHCA, timely initiation of basic life support (BLS) by lay bystanders is crucial to ensure a timely perfusion of the brain and other vital organs. Sufficient perfusion can only be obtained by high-quality CPR. Hence, it is vital to increase the percentage of bystander-CPR and to improve the quality of both BLS and ALS. According to the German Resuscitation Registry, bystander-CPR is performed in less than 20 % of OHCA cases [16], as opposed to 50 % or more in Scandinavian countries. Large-scale programmes raising public awareness of cardiac arrest and CPR as well as providing hands-on training in BLS are a promising tool to improve survival after OHCA. In a national initiative that included CPR-training as early as in primary school, telephone guidance to CPR by EMS-dispatchers, and distribution of automated external defibrillators (AED) in Denmark [17], the rate of bystander-CPR could be increased from 21 to 45 % and 1-year survival raised from 3 to 10 % during the intervention period. It is worth noting that the percentage of AED-use increased only from 1 to 2 %, which implies that the impressive improvement of outcomes of OHCA patients is rather due to prompt basic life support (as well as improved post-resuscitation care) than to AEDs. Similar initiatives are now being undertaken in Germany. Once CPR is started, its high quality is decisive for outcome. Compression depth must be sufficient (5–6 cm), frequency appropriate (100–120/min.), the chest must be released between compressions and interruptions of chest compressions must be kept as short as possible. Several studies have shown an association between these factors and survival [18–20]. During ALS by EMS-personnel, further variables are part of CPR quality: in the OHCA-setting, automated chest compression devices have failed to prove superiority to manual CPR. There is still much debate on airway management and staffing of ambulance cars. Retrospective studies have shown higher survival rates in patients who received endotracheal intubation (ETI) as compared with extraglottic airways [21, 22]. In retrospective studies, though, successful ETI might also be indicative of a generally higher skill-level of the EMS-personnel performing CPR. When using ETI during CPR, one needs to keep in mind that a high level of training is indispensable and that prolonged ETI-attempts, which go along with long interruptions of chest compressions, must be avoided. In a recent prospective, non-randomized trial, presence of a physician during CPR of OHCA-patients was associated with an impressively higher rate of survival than in patients being resuscitated by paramedics only [23], which confirms results of previous trials that observed higher survival rates in physician-staffed EMS [24]. Taken together, we can save thousands of lives each year if we manage to increase the likelihood of bystander-CPR and to optimize quality of ALS.

#### What to perform prior to ICU admission? Percutaneous coronary intervention before hypothermia

##### Hendrik Drinhaus and Bernd Böttiger

Mild therapeutic hypothermia (MTH) and percutaneous coronary intervention (PCI) are both established components of post-resuscitation care after cardiac arrest, as recommended by the 2008 Statement of the International Liaison Committee on Resuscitation (ILCOR) [25]. Already then, it was suggested that indication for early PCI be extended beyond obvious myocardial infarction (STEMI), given the high probability of significant coronary artery disease in OHCA patients [26]. Since then, several trials have underlined the beneficial effects of early (<6 h after the event) PCI after OHCA also without STEMI [4, 27, 28]. Combination of PCI and MTH is feasible and does not necessarily lead to longer door-to-balloon times [29]. Its effects on survival and good neurological outcome appear to be synergistic [30, 31]. Hence, modern post-resuscitation care includes hypothermia and early coronary intervention (unless a non-cardiac origin of cardiac arrest is assumed or confirmed). Induction of MTH is recommended to be started as soon as possible by the ERC guidelines. Meta-analyses of the existing data on very early and prehospital induction of MTH have not shown an improvement in survival [32]. In a recent large randomized controlled trial using large volumes of ice-cold saline to induce MTH before arrival at the hospital, no difference in survival or neurological outcome could be found, but patients who received cold saline had a slightly higher risk of renewed cardiac arrest during transport to the hospital and of transient pulmonary oedema [33]. In the light of the findings presented hitherto, we deem it important to stress the survival benefit of early PCI after cardiac arrest not only due to definite STEMI and to raise awareness among EMS personnel to transport OHCA-patients to hospitals in which early PCI, as well as other components of post-resuscitation care, can and will be performed at any time. Prehospital cooling (at least by infusion of cold saline) on the other hand appears not to convey a benefit, maybe even a risk, and hence, not too much time and effort should be spent on aggressively lowering temperature during transport using ice-cold saline solution. In any case, induction of MTH must not delay arrival at an appropriate hospital. It appears worth considering to include into the guidelines on resuscitation and post-cardiac arrest care a recommendation to treat patients after cardiopulmonary in specialized centres that can provide early PCI, MTH and targeted temperature management as well as expert ICU-treatment wherever and whenever possible [34].

#### What to perform prior to ICU admission: is CT-scan useful?

##### Alain Cariou

**Fig. 4:**
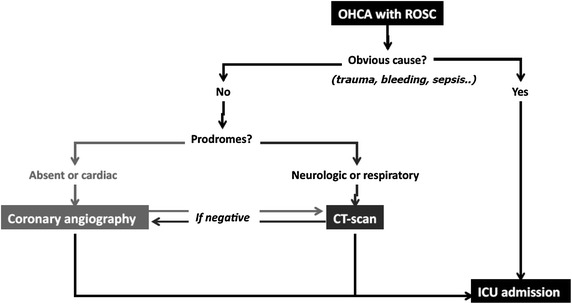
Algorithm for early imaging diagnosis after cardiac arrest

Early identification of causes and consequences of cardiac arrest is generally considered of importance, in order to prevent recurrence and subsequent clinical deterioration. Apart from coronary angiography, a brain and chest CT-scan can also be performed at admission, when an extra-cardiac cause is suspected and in the absence of an obvious pre-hospital etiology. In a recent study, this strategy was performed in 355 patients and provided a diagnosis in 72 patients (20 %), mainly stroke and pulmonary embolism (PE) [35]. Early identification of brain damages can lead to major changes in therapy such as the use of anticoagulants. In addition, since the rate of subsequent brain death is very high in this subgroup, organ donation can be considered rapidly if an early diagnosis is achieved. Regarding PE, current guidelines underline the potential benefit of identifying this curable cause of arrest [36]. Based on prodromes and clinical evidence, an algorithm can be proposed in order to manage early imaging after cardiac arrest (Fig. 4).

#### Thrombolysis in the treatment of cardiac arrest

##### Hendrik Drinhaus and Bernd Böttiger

In non-traumatic OHCA, acute myocardial infarction (AMI) and pulmonary artery embolism (PAE) account for approximately 70–80 % of all cases [37]. Common feature of these aetiologies is the obstruction of vital arteries by a blood clot. Dissolving these blood clots by administering fibrinolytic substances to re-establish circulation in the respective vessel beds therefore appears to be a logical approach from a pathophysiological point of view. Several case-reports and case-series have shown impressive results in patients with presumed or documented PAE [38, 39]. Improvement of return of spontaneous circulation (ROSC) and survival rates in patients who received thrombolysis during CPR has also been observed in non-randomised observational trials [40, 41]. A large randomised, double-blind, placebo-controlled trial—the TROICA-study—was therefore initiated to systematically evaluate thrombolysis during OHCA of presumed cardiac origin, not limited to suspected pulmonary artery embolism. In this trial, no difference with regard to ROSC or survival rates in an unselected OHCA-population could be detected. Intracranial haemorrhage occurred more frequently in the tenecteplase-group [37]. Hence, no recommendation to administer fibrinolytics as a standard of care in all OHCA-patients could be generated from this trial. Accordingly, the current guidelines of the European Resuscitation Council recommend not to use fibrinolytics routinely during CPR. However, fibrinolytics should be considered if pulmonary embolism is the proven or suspected cause of cardiac arrest. In this case, cardiopulmonary resuscitation should be maintained for at least 60–90 min after injection of the fibrinolytic, if necessary [42]. Taken together: if a coronary cause of cardiac arrest is assumed, fibrinolytic drugs should not be used and prompt coronary revascularisation, usually by percutaneous coronary intervention, is the treatment of choice—even during ongoing CPR. Fibrinolytics must be considered if pulmonary artery embolism is the suspected or proven cause of cardiac arrest.

#### CPR is better without epinephrine in cardiac arrest!

##### Kjetil Sunde

Whereas there is clear evidence for improved survival with CPR and defibrillation during cardiac arrest management, there is today lacking evidence that any of the recommended and used drugs leads to any long-term benefit for the patients. Thus, until we have better drugs or combination of drugs, ALS can be performed without the use of drugs, and instead gains all focus on improving the tasks we know improve survival. Good-quality CPR, early defibrillation together with goal-directed post-resuscitation care is way more important than giving drugs with no proven benefit [43]. More drug studies are indeed required, and future research needs to incorporate better diagnostic tools to test more specific and tailored therapies that account for underlying aetiologies and individual responsiveness. We should expand our diagnostic capabilities exploring the feasibility of utilizing technologies such as capnography, near-infrared spectrophotometry (NIRS), VF analysis, and ultrasound assessment to allow targeted therapy (while maintaining adequate CPR). When good quality of care and improved diagnostics have been ensured, more tailored drug approaches could eventually be tested based on underlying etiologies.

#### ECMO for cardiac arrest: ECMO is futile

##### Martin Dworschak

Extended time periods of no and low flow after CA are generally associated with poor outcome. Although return of spontaneous circulation can be achieved after more than 20 min of CPR, only few patients will survive with good functional outcome [44, 45]. By rapid deployment of ECMO in patients with refractory conventional CPR (CCPR) systemic blood flow can be maintained to prevent irreversible organ damage. The best results with extracorporeal CPR (ECPR) have been obtained so far in neonates and children when ECPR was instituted during in-hospital cardiac arrests that had short response times. The benefit of ECPR in adults being resuscitated for in- or OHCA is less clear. One major confounder is the fact that ECPR is frequently considered for a highly selected patient population (young age, ventricular fibrillation as the initial cardiac rhythm, witnessed arrests with immediate bystander CPR) only [46]. Nevertheless, quoted survival rates after ECPR [47] are either comparable with those in CCPR patients [48] or slightly better, but this does not seem to impact on neurological outcome [46, 49]. Accordingly, in 2010 the American Heart Association did not recommend the routine use of ECPR [50]. Yet, it could be taken into consideration when it is readily available and the no-flow duration is brief. Furthermore, the conditions that led to the arrest should either be reversible or amenable. Particularly in OHCA, however, most patients are neither young nor hypothermic or intoxicated and they do not present with a shockable rhythm; fewer CAs are witnessed and the quality of BLS/ALS is usually unknown. Another handicap in OHCA appears to be fast deployment of ECMO with quick institution of therapeutic hypothermia [51]. Although ECPR seems to improve survival, especially after long-duration CPR, the rate of neurologically intact survivors still remains low. Future research should define criteria for the optimal indication of ECPR and criteria for ECPR as a bridge to LVAD, HTX, or organ procurement to guarantee efficient use of precious and scarce resources. Randomized controlled trials, as well as crucial analysis of uniformly reported CA data from registries would greatly facilitate decision-making.

### Post-resuscitation management: targeted temperature management

#### How to cool?

##### Fabio Taccone

Several cooling methods, both invasive and non-invasive, are currently used to achieve target temperature after post-anoxic brain injury. Invasive methods include the administration of intravenous cold fluids or the use of endovascular cooling catheters [52]. The use of cold (4 °C) fluid infusion is cheap and easy-to-use, even in the pre-hospital setting. This method is generally recommended to initiate cooling in comatose survivors after CA, either alone or in conjunction with other cooling systems [53]. A 2-l bolus given over 30–60 min immediately after ROSC was associated with a mean decrease in core temperature of 1.3 °C [54]. This method is largely used also to induce hypothermia in the fields [33]. However, cold fluids are not effective to maintain target temperature [55] and, because of potential excessive volume-loading and reduced coronary perfusion pressure [56], have been recently associated with an increased risk of re-arrest and pulmonary oedema when given before hospital admission [33]. The endovascular cooling catheter contains a circulating cold solution, which is maintained at a controlled temperature; this method can easily achieve a cooling rate of 1.5–4.5 °C/h [52]. Moreover, the use of an endovascular system reduced the variability of body temperature around the target value and increased the proportion of time that patients spent within the therapeutic temperature targets during the maintenance phase [57]. Main limitations are related to the time to insertion into a large vein, the need of a bedside heat exchanger with energy supply (i.e. limited used outside the ICU) and the potential risk of venous thrombosis or infection [58].

Non-invasive methods include external surface and ice packs or pads. Modern cooling blankets or fluid pads usually operate with a continuous temperature feedback mechanism, which reduced the risk of temperature variability and overcooling, and present a cooling rate around 1.2–2.5 °C/h in CA patients [52, 59]. Unfortunately, these devices also depend on an external energy supply and it remains difficult to use them outside the ICU. Ice packs can be easily applied to different areas of the body, are inexpensive and are not dependent on an external energy source; however, the cooling rate is extremely poor (<1 °C/h) and could expose patients to overcooling, as there is no feedback temperature control. Ice pads can provide a faster cooling rate (up to 3 °C/h) after CA, but they have been associated with thermal skin damage on the sites of application [60].

An attractive alternative to these methods is to induce brain hypothermia, especially because of the decreased risk of side effects associated with whole-body cooling. Cranial cooling cap devices placed around the head and neck can easily decrease tympanic temperature; however, they are mostly effective in children because of their favourable ratio of head-to-body surface area [61]. Nasopharyngeal cooling devices, which use volatile coolant fluids via a specific nasal catheter, can rapidly reduce brain temperature with a concomitant reduction of core temperature around 1.0–1.5 °C/h [62]. These systems are quite safe, are not dependent on an external energy source and can be initiated even during cardiopulmonary resuscitation [63]. Alternatives methods for cooling could be continuous renal replacement therapy, peritoneal lavage and, in case of severe cardiogenic shock, the use of extracorporeal membrane oxygenation (ECMO) devices; however, these methods are particularly invasive and their use has been limited to very selected cases [64–66].

Finally, no prospective study has shown a clinical superiority of one method to another, while some retrospective reports suggest a better survival rate for endovascular cooling when compared to surface cooling methods [67, 68]. Importantly, the endovascular catheter and modern surface devices have significant higher costs than intravenous fluids and ice packs [69]; however, their use was associated with an important reduction of nurse workload and improvement in patients’ care [70].

#### For how long?

##### Kjetil Sunde

**Fig. 5:**
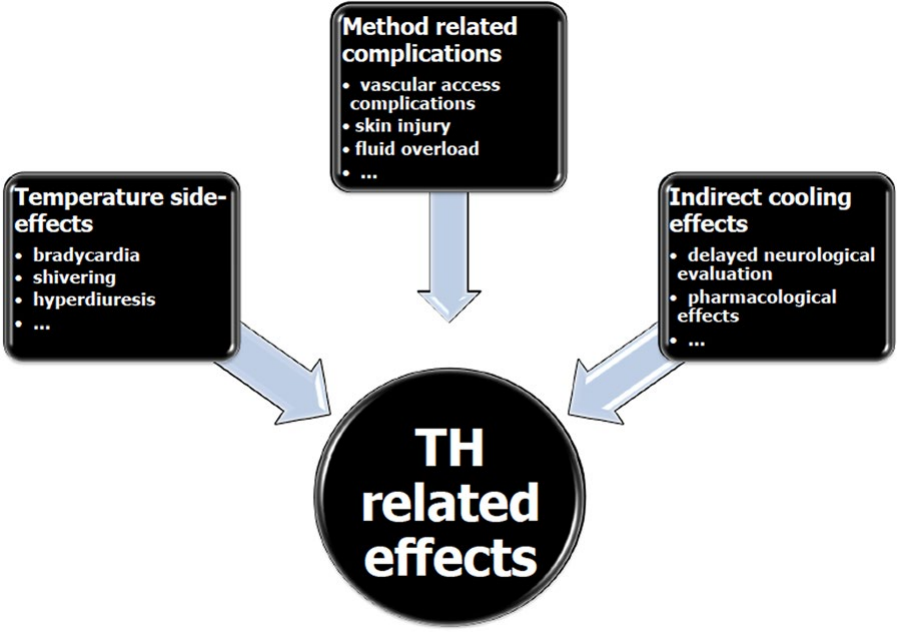
Therapeutic hypothermia side-effects and their categorization

The precise description of patients that will benefit from therapeutic hypothermia (TH), or targeted temperature management (TTM), the ideal induction technique (alone or in combination), target temperature, optimal maintenance and rewarming times have yet to be established. Independently of the cooling method chosen, TH is easy to perform, and without severe side effects or complications associated with mortality. As for the duration of TH/TTM, the majority of centers use 24 h as their treatment strategy; however, we are lacking of clinical data comparing different durations. Newborn asphyxial arrests are treated with TH for 72 h, with several RCTs proving its benefit compared to normothermia/fever. In a recent animal study, post-cardiac-arrest TH resulted in comparable improvement of survival and survival with good neurologic function when initiated within 4 h after return of spontaneous circulation. Interestingly, histological assessment of neuronal survival revealed a potentially broader therapeutic window and greater neuroprotection when TH was maintained for 48 versus 24 h [71]. However, this benefit was not seen in the neurological outcome evaluation. In a clinical registry study among approximately 1000 cooled comatose cardiac arrest patients, factors related to the timing of TH had no apparent association to outcome (Fig. 5).

#### Any evidence for non-shockable rhythm patients?

##### Nicolas Deye

According to international guidelines, therapeutic hypothermia (TH) might also benefit comatose adult CA patients with spontaneous circulation after resuscitation from a non-shockable rhythm [72]. However, there is no large randomized controlled trial evaluating the clinical impact of TH in this situation. Using TH for non-shockable CA patients is supported by only low level of scientific evidence and extrapolation of data resulting from shockable rhythms. Consequently, the potential impact of TTM including TH-implementation remains controversial for CA patients presenting with initial non-shockable rhythm [73, 74]. Treating such patients becomes a major health issue, as the proportion of these patients increases over decades while the proportion of OHCA patients resuscitated from shockable rhythms declines and now represents the minority of OHCA patients [75]. Prognosis of patients experiencing CA still remains poor. However, large registries reported recently for non-shockable CA patients hospitalized in ICU survival up to 26 %, favourable neurological outcome at 6 months of 22 %, and survival rate at 10 years after hospital discharge alive reaching 43 % [76, 77].

The pro-con debate on the use of TH in non-shockable patients has been previously described [74]. Briefly, some argue in favour of the use of TH in non-shockable CA patients: (1) the pathophysiological processes responsible of post-anoxic (brain) damages seem independent of the initial rhythm (i.e. shockable versus non-shockable); (2) most of experimental and animals studies strongly support the use of TH in asphyxia and CA models irrespective of the initial rhythm; (3) with more than 10 concordant large randomized controlled trials and meta-analyses, TH initiated within 6 h and targeted to 32.5–35.5 °C for 72 h is unequivocally beneficial on survival and neurological outcome in newborns who sustained asphyxia (with or without cardiopulmonary resuscitation: CPR) and exhibit acidosis and/or neonatal hypoxic-ischaemic encephalopathy [73, 74, 78]; (4) TTM with TH is potentially the unique available treatment to date prone to minimize brain damages and long-term disabilities, and to increase survival without major sequelae in non-shockable CA patients [74]; (5) some observational, retrospective or prospective, but non-randomized clinical studies observed a benefit of TH in non-shockable CA adults patients, with one meta-analysis suggesting a reduced in-hospital mortality without improvement of the neurological outcome in these patients.

Conversely, the potential increased TH-related side effects are not in favour of the use of TH in non-shockable CA patients. Adverse events include the increased delay in time to recovery of consciousness leading to a prolonged delay to evaluate the neurological recovery. This delay seems mainly induced by the use of sedatives and neuromuscular blockers often required during the TH use, leading to a possible prolongation of mechanical ventilation and hospitalization durations, and consequently of ICU costs. The increased incidence of other main side effects, essentially pneumonia and sepsis, could be another explanation altering the risk/benefit ratio of TH, although neither firmly established nor related to the initial CA rhythm [74, 76, 79].

Additionally, several studies describing non-shockable CA patients have not shown significant prognostic effect of TH [74]. Most of the studies included in the first meta-analysis evaluating this issue presented substantial risks of bias and a very low quality of evidence. Thus the beneficial effect observed for in-hospital mortality was no longer significant when analysis was restricted to the two small RCTs available. Another meta-analysis similarly concluded that the group sizes for patients with asystole or non-cardiac causes of CA were too small to draw conclusions. At least, several recent non-randomized controlled studies—after adjustment in some studies—reported negative or even harmful TH-effects on outcome in this population [74, 80–82]. Similarly, with a TH implementation in nearly 50 % of patients and a global hospital survival of 2 % in the non-shockable rhythm group, TH was not significantly associated with hospital survival after adjusting for other prognostic factors [75].

Beyond ethical considerations regarding the TH-related futility/benefit ratio, some major issues remain unsolved regarding the ideal TTM in this specific population.

The correct selection of patients that could benefit or not from TTM or TH-implementations seems critical in this heterogeneous group, as well as the underlying co-morbidities and severity of the post-resuscitation syndrome [74]. Indeed an initial non-shockable rhythm may represent: (1) either the first documented CA rhythm resulting from a severe non-cardiac aetiology with its own prognosis per se; (2) or the consequence of an initial shockable CA with a prolonged time to first CPR leading to prolonged ischaemic times, severe brain damages and/or multiple organ failures. A non-prolonged time from collapse to return of spontaneous circulation (i.e. sum of the time to first CPR plus the duration of CPR) could be a relevant prognosis factor able to identify patients who may mostly benefit from cooling independently of the initial rhythm. Conversely in witnessed-OHCA patients, the more prolonged is the time from collapse to first CPR the better could be the TH-benefit after adjustment on other factors including the initial rhythm. The target population in non-shockable patient probably results from a complex balance between patients that can benefit from TH (i.e., those with TH-accessible brain damages with adequate durations of time to first CPR and CPR durations) versus those who do not (i.e., those with multiple organ failure or too severe prognosis that could lead to early death or early treatments withdrawal). Additionally, the TH-implementation in non-shockable patients is often left at the bedside physician’s discretion in most studies evaluating this issue, and important bias should be introduced regarding the correct selection of patients [74, 83].

At last, the aetiology and/or mechanisms responsible for fever are possibly a key issue to correctly evaluate patients able to receive a TTM or a TH implementation, especially after CA occurrence in non-shockable patients. In a large population of unselected ICU patients, fever was not always associated with a poor prognosis and control of fever was not always associated with better prognosis, with important differences existing between septic and non-septic patients [84]. Post-CA syndrome often mimics a sepsis-like syndrome. The correct discrimination of patients who may benefit for a strict normothermic control, those who may benefit for a TH implementation targeted to 32–35 °C, versus those who may—or not—benefit for a fever control within the 48–72 h after CA to avoid rebound hyperpyrexia, is the next step to optimize the temperature control after CA in the non-shockable population [74, 85].

The choice of the correct target temperature as the precise achievement of goal temperature seems also crucial in these patients. The recommended treatment for all post-CA patients is at present an early TH-implementation of 12–24 h targeted to 32–34 °C [72]. An adapted scheme of TTM could be of critical importance in non-shockable patients, regarding the optimal duration, speed cooling, level of temperature, therapeutic window, and rewarming rate of TTM, considering that the cerebral damages may be more severe in this population [72, 74]. Interestingly, in the large recent TTM-trial including 20 % of patients presenting with an initial non-shockable rhythm out of a total 939 enrolled patients, no difference were found between the 2 studied arm, i.e. 33 versus 36 °C applied for 28 h. The TTM-scheme included in both groups a gradual rewarming <0.5 °C/h to reach 37 °C, with tapering or discontinuation of the mandatory sedation at 36 h, and a maintenance of body temperature for unconscious patients <37.5 °C until 72 h after CA [86]. Similar results were herein observed in all pre-planned subgroups including analyses performed according to initial rhythm (i.e. non-shockable versus shockable), suggesting that “strict normothermia” targeted to 36 °C should be an alternative in non-shockable rhythm CA patients.

This ongoing debate clearly underlines the need for a large multicentre study evaluating the effects of different scheme of TTM and TH in non-shockable CA patients after careful patients’ selection, including insights in subgroups according to the pathophysiology of the CA. To date, the indication for TH in non-shockable patients should be based using a case-by-case approach, whereas a TTM approach targeting strict normothermia remains reasonable for these patients.

#### Hypothermia-associated complications

##### Alain Cariou

Besides infections, several other adverse events such as arrhythmias, seizures, bleeding or thrombosis, electrolyte and metabolic disorders, occur commonly in comatose patients treated in critical care units after out-of-hospital cardiac arrest. These events may be related to the cause precipitating the cardiac arrest, the post-cardiac arrest syndrome or the critical care treatment. As it may affect many physiologic processes and responses, hypothermia itself is commonly suspected to promote these events (Fig. 2) [87]. However, most of these events are probably not related to TH, as suggested by the results of a recent Cochrane systematic review that revealed no significant difference in reported adverse events between hypothermic and control patients [88]. Regarding metabolic disorders, rapid changes in glycemia are possibly the most clinically relevant event that could be worsened by hypothermia [89, 90]. As it may worsen brain damages, it suggests minimizing glycaemic variations during the post-resuscitation period. In addition, TH most often requires sedation, ventilation, and neuromuscular blockade, which may delay the possibility of neurological evaluation.

#### What is a real targeted temperature management: TTM at 36 °C

##### Hans Friberg

In 2002, two landmark trials were published showing that lowering body temperature to 33 °C improved neurological outcome and saved lives in comatose survivors of out-of-hospital cardiac arrest [52, 91]. This therapy was rapidly adopted by international guidelines and not questioned until a systematic review using the GRADE methodology and trial sequential analysis showed that the quality of the evidence for treating cardiac arrest patients at 33 °C was low [86]. Criticism included the low number of enrolled patients and the fact that both trials had weaknesses, including quasi-randomization, lack of power analysis and early stopping which was not adjusted for. Furthermore, patients in the control groups were treated according to standard care of that time which allowed fever [92].

**Fig. 6:**
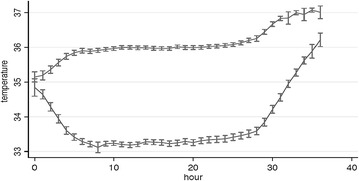
Mean bladder temperature in the 33 and 36 °C intervention groups of the Target Temperature Management after Out-of-hospital Cardiac Arrest Trial (TTM-trial), during the 36 h of temperature intervention. Temperature values are presented with 95 % confidence intervals. In the original publication, temperature curves displayed the means ± 2SD. From Wise MP et al. [179]. Reprinted with permission

This led to the design of a new trial, randomizing patients to two controlled temperatures, 33 and 36 °C [93]. The Target Temperature Management after Out-of-hospital Cardiac Arrest trial (TTM-trial) included and randomized 950 patients in 27 months, almost three times the number of the two previous trials combined. Thirty-two sites enrolled patients in nine countries in Europe plus Australia. The TTM-trial differs from previous trials in several aspects, the most important one being that all patients received controlled temperature management avoiding fever in both intervention arms (Fig. 6). Other differences include a contemporary setting with improved pre-hospital care with a high bystander CPR rate, and improved hospital care in patients with ROSC. For example, two of three patients in the TTM-trial received an early angiography (<24 h). Other quality criteria that were acknowledged in the TTM-trial, as opposed to previous trials, were that the design and the statistical analysis plan were published in advance. [93, 94] In addition, prognostication of comatose patients was performed according to a strict and predefined protocol and the rules for decisions on withdrawal of life-sustaining therapy (WLST) were transparent and published in advance [90]. The primary outcome was survival until the end of trial. A neurological assessment, using both the Cerebral Performance Category scale (CPC) and the modified Rankin Scale (mRS), was performed at 6 months and was blinded.

**Fig. 7:**
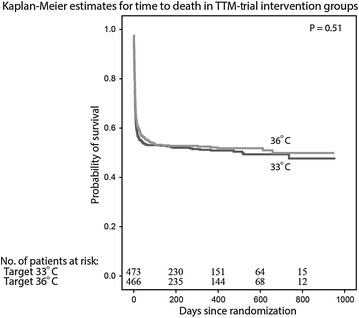
The TTM-trial included and randomized 950 comatose patients to either 33 or 36 °C. Probability of survival through the end of the trial and the number of patients at risk at each time point is presented. From Nielsen et al. [86]. Copyright © (2013) Massachusetts Medical Society. Reprinted with permission

In summary, the TTM-trial has sent a clear message to the medical community that no difference in survival (Fig. 7) or neurological outcome could be detected when comatose patients after cardiac arrest were treated at either 33 or 36 °C [86]. We can therefore conclude that a real target temperature management after cardiac arrest is a controlled temperature at either of the two temperatures, both avoiding fever. This should be part of a bundle of care, including treatment of acute coronary disease and contemporary and active intensive care.

Our rationale for changing to 36 °C is that this temperature is closer to normal and less invasive, reducing known and unknown risks. The number and severity of adverse events in the two intervention arms did not differ in the TTM-trial, although there was a tendency towards less events in the 36°-arm (*p* = 0.086). Treating the patients closer to normal temperatures allows for predictable drug effects, including those of sedatives and antithrombotic therapy. In a recently published post hoc analysis of the TTM-trial of the subgroup of patients in circulatory shock at admission, a higher mortality at ICU discharge was suggested in the 33 °C-arm (66 versus 44 %, adjusted *p* value 0.03) [95]. We cannot exclude that other subgroups may benefit from treatment at 36 °C or for that matter, from treatment at 33 °C. The optimal temperature, duration of temperature management and target population are yet to be defined.

### Post-resuscitation management: optimizing organ perfusion and metabolic parameters

#### Oxygen and carbon dioxide after cardiac arrest: friend or foe?

##### Nicolas Deye

In CA patients, neurological injury is a major cause of mortality [72]. A first hypoxo-anoxia phenomenon occurs initially during CA before and during CPR maneuvers. The ischemia–reperfusion syndrome occurring in the post-resuscitation phase after obtaining an ROSC can lead to secondary insults including oxidative stress with free radical formation or mitochondrial dysfunction [72, 96]. Oxygen (O_2_) and carbon dioxide (CO_2_) abnormalities can promote these insults. Targeted temperature management (TTM), including therapeutic hypothermia (TH) targeted to 32–34 °C, is one of the therapeutic measure improving prognosis and neurological outcome after CA. Induced hypothermia could minimize the CA-related injuries by decreasing O_2_ free-radical production, mitochondrial dysfunction, brain O_2_ consumption [96]. Thus TH could minimize neuronal death and improve neurological outcome and survival. However, hypothermia is associated with several arterial blood gas (ABG) modifications, mainly induced by the leftward shift of the hemoglobin dissociation curve, increased O_2_ and CO_2_ solubility, modification of the pH regulation, with hypoventilation and hypometabolism, leading per se to hypoxia and hypocapnia. Iatrogenic dyscarbia incidence (hypo- or hyper-carbia) has been reported up to 69 % [97], hypoxia up to 63 % [98], and hyperoxia up to 41 % [99]. International guidelines mainly focused on potential hypocapnia and hypoxia/hyperoxia harmful effects for CA patients [72, 100]. They advocate initial resuscitation with 100 % O_2_ ventilation to avoid hypoxia followed by a titration of O_2_ therapy targeting arterial O_2_ saturation levels between 94 and 96(−98) % especially during the initial post-CA period to avoid hyperoxia.

Beyond general detrimental effects such as decreased stroke volume, cardiac output and coronary blood flow and increased systemic vascular resistances, potential neurological harm of O_2_ therapy have been largely described [100, 101]. Hyperoxia can exacerbate cellular oxidative stress injury and mitochondrial dysfunction to key mitochondrial enzymes or mitochondrial lipids, leads to cerebral O_2_-related vasoconstriction with a decreased cerebral blood flow (CBF) and cerebral energy metabolism impairment. It can increase the neuronal lipid peroxidation and protein oxidation, enhance O_2_ free radical formation and reactive O_2_ species, or react with nitrite oxide to produce toxic metabolites (peroxynitrite, superoxide ion, hydrogen peroxide). All of this will finally participate to cell death. Despite several limitations or controversial results in specific experimental models [100–102], most animal studies have suggested that hyperoxia after CA could worsen neurological outcome [103].

In a retrospective cohort including 145 adult OHCA patients with available arterial O_2_ pressure (PaO_2_) on ABG sample during CPR attempts, an increased PaO_2_ was associated with improved rate of hospital admission alive in multivariate analysis [104]. Incidence of hyperoxia defined as PaO_2_ >300 mmHg reached 14 %, whereas hypoxia was defined as PaO_2_ <60 mmHg. The potential deleterious effect of hypoxia during the resuscitative efforts has been also found in another recent retrospective study [100].

Most clinical studies depicting the O_2_ effects after ROSC from CA are methodologically of low level quality. The only small randomized clinical trial evaluating this issue found a significant decreased value of the Neuron Specific Enolase biomarker in favor of a normal level of PaO_2_ versus a high level PaO_2_ in the subgroup of patients without TH [105]. However, this result was not observed for the S100B Protein biomarker and in the whole cohort including the 28 patients regardless TH implementation. Hypoxia and hyperoxia harmful effects regarding in-hospital mortality and functional status were initially described in a large American registry in 2010 [98]. Several issues have been pointed out for these studies: (1) the statistical methods (registries and databases versus scarce randomized studies, retrospective versus prospective studies, single center versus multicentre studies, adjustment not usually performed to control for other potential confounders); (2) the definitions regarding hyperoxia thresholds (i.e. what precise level of PaO_2_ to choose?); (3) the different time-point measurements and the period of data collection (i.e. what value to consider between the first ABG, the ABG on admission, the ABG within the first hours or the first day after ROSC or CA, between the mean or median versus the maximal or minimal PaO_2_ values or the worst PaO_2_ using worst (A-a) DO_2_ or the PaO_2_/FiO_2_ ratio?); (4) the associated treatments (i.e. TH versus the absence of TTM); (6) the best endpoint (i.e. in-hospital mortality versus neurological outcome).

A recent meta-analysis concluded that hyperoxia in the post-resuscitation phase after ROSC was significantly associated with an increased in-hospital mortality [99]. Conversely, the poor neurologic outcome at hospital discharge did not reach significance suggesting a possible lack of association or a review underpowered. However, these results need further confirmation because of its significant heterogeneity (results were inconsistent in subgroup and sensitivity analyses) and the limited number of studies analyzed (3 abstracts out of the 10 pooled studies were finally included). Since this review, two other studies describing a low hyperoxia incidence rate (3 and 6 %) have been published [106, 107]. The potential harmful effect of hyperoxia regarding mortality was not significant. In only one of these 2 studies [106], hyperoxia was significantly associated with poor neurological outcome in the multivariate analysis, depicting a “V-shaped” relationship between probability of unfavorable outcome and the mean PaO_2_ value obtained from ROSC to rewarming.

Cerebral autoregulation physiologically maintain a constant CBF within a large range of mean arterial pressure [100, 108]. This relationship is modified by dyscarbia as cerebral perfusion depends on CO_2_. Hypercapnia leads to cerebral vasodilation and potentially increased intracranial pressure, whereas hypocapnia leads to cerebral vasoconstriction and potentially ischemia (decreasing PaCO_2_ of 1 mmHg can decrease CBF up to 3 %). Ensuring physiological CO_2_ tension in CA patients seems important to prevent worsening of the neurological status. Furthermore, impaired autoregulation has been described in some brain-injured areas, in some TH-treated patients, and inconstantly in CA patients. Recommendations in resuscitated CA patients suggest a PaCO_2_ target of 40–45 mmHg during the post-ROSC period in this population of brain-injured patients regardless of TH use [72, 100]. In a retrospective single-center study implementing TH in 41 %, hypocapnia (defined by PaCO_2_ ≤30 mmHg), hypercapnia (PaCO_2_ ≥50 mmHg), and association of both dyscarbia occurring within the first 24 h after ROSC were all independently associated with poor neurologic outcome at hospital discharge [97]. An observational multicenter registry recently enrolled 16.542 adult CA patients TH-treated in 39 %. Increased in-hospital mortality and rate of poor outcome were observed in the hypocapnia group compared to normocapnia after adjustment for illness severity and propensity score [109]. Conversely, hypercapnia (PaCO_2_ ≥45 mmHg) within the first 24 h after admission was independently associated with similar in-hospital mortality and a higher rate of discharge home among survivors. In a smaller study of TH-treated patients, hypocapnia but not hypercapnia was independently associated with an increased risk of in-hospital death [106]. A “U-shaped” relationship between the mean PaCO_2_ and the in-hospital mortality was found with the best survival observed for the normocapnia group (35–45 mmHg). No association between hypo- and hyper-capnia with poor neurologic outcome were observed. Another recent multicenter and prospective study applying TH in 71 % defined PaCO_2_ as “low” when <30 mmHg, “middle” when 30–37.5 mmHg, “intermediate” when 37.5–45 mmHg, and “high” when >45 mmHg [107]. Patients with poor versus good outcome had similar highest, mean and lowest PaCO_2_. The mean 24-h PaCO_2_ and the time spent in PaCO_2_ >45 mmHg regardless TH-implementation were independently associated with better 1-year good outcome. However, same criticisms can be made for all these studies than those made above regarding statistics, threshold, data collection, or treatments. To date mainly because of paucity of data on this issue, no clear thresholds have been found regarding the harmful impact of hypo- or hyper-capnia after CA. Whereas hypocapnia seems consistently associated with worse outcome, hypercapnia is not [100]. The management of PaCO_2_ after CA by using specific mechanical ventilation strategy could influence the outcome of these patients especially during TH. In a recent retrospective study focusing on initial post-CPR mechanical ventilation settings minute ventilation was weakly correlated with the initial PaCO_2_ [110]. Normocapnia alone (31–49 mmHg) was again associated with a better favorable neurological outcome.

ABG measurements performed at 37 °C are secondarily expressed either as temperature-corrected or uncorrected according to biochemical centers [108]. Substantial discrepancies can be related to these methods: PaO_2_ = 100 mmHg at 37 °C becomes 79 mmHg if corrected at 33 °C, when PaCO_2_ = 36 mmHg becomes 30 mmHg at 33 °C. There are no clear recommendations regarding the ventilation strategy to be used in resuscitated CA patients despite such differences. Normocapnia in hypothermic patients can be achieved according to two different mechanical ventilation strategies: α-stat versus pH-stat. In the α-stat strategy ventilation is set to achieve physiological arterial CO_2_ tension measured at 37°, unadjusted to the patient’s temperature, whereas in the pH-stat strategy ventilation is set to achieve physiological arterial CO_2_ tension measured at the patient’s actual temperature. The latter strategy leads to a relative hypoventilation compared to the α-stat strategy. Using either α-stat or pH-stat strategy to guide normocapnia after CA remain open to discussion. In the recent multicenter Finnish study focusing on ABG abnormalities after CA, 13 ICUs used temperature-correction; whereas, eight did not [107]. Two recent exploratory studies in TH-treated CA patients compared either a “lower versus upper threshold normocapnia” (32 versus 45 mmHg) or the α-stat versus the pH-stat strategies while maintaining a PaCO_2_ target level between 36 and 42 mmHg. The first study showed that the “lower threshold normocapnia” induced decreased internal jugular vein O_2_ saturation and CBF mean velocity suggesting an increased risk of cerebral ischemia. The second study found that the alpha-stat strategy increased jugular vein desaturation and cerebral O_2_ extraction and decreased transcranial Doppler cerebral velocities in survivors but not in non-survivors.

In summary, dyscarbia especially hypocapnia should be associated with an increased harm after CA leading to increased unfavorable outcome or in-hospital mortality. Conversely O_2_ seems a two halves phenomenon with: (1) a former deleterious effect of hypoxia during resuscitation efforts, the potential benefit of hyperoxia implying more evaluations; (2) followed by a deleterious effect of both hypoxia and hyperoxia after ROSC in the post-CA phase. To date because O_2_ and CO_2_ derangements have no obvious benefit, aiming at normoxia and normocapnia after CA are potentially two very easy targets to achieve in post-resuscitation care bundles. Meanwhile all studies describing ABG parameters in TTM- or TH-treated CA patients should actually emphasize the strategy used, either a temperature-corrected or a non-corrected strategy to more precisely evaluate potential thresholds.

#### Other means of cardio- and neuro-protection

##### Fabio Taccone

Heart and brain protection strategies aim to prevent or attenuate disease progression and secondary injuries by halting or at least slowing the loss of cardiomyocytes and/or neurons [111]. Although many pathological mechanisms, including endothelial damage and tissue hypoperfusion, inflammation, impaired mitochondrial respiration with induction of reactive oxygen species, calcium overload or excitotoxicity, are common in both cardiac and neurological injury following CA [112], some drugs may present a more specific organ protection or have a lower brain penetration, thus acting especially on the cardiac tissue. Many therapeutic agents have been shown to be effective in protecting the heart and/or the brain in animal models of global or local ischemia [113]; nevertheless, these findings were flawed because of the differences between the experimental setting and the clinical scenario (i.e. absence of co-morbid diseases and need for anesthetics in animal studies), the low mortality rates or the administration of the specific drug before the development of injury (i.e. pre-treatment approach).

A promising approach to reduce brain excitotoxicity (i.e. excessive extracellular glutamate levels) could be the administration of intravenous magnesium or inhaled noble gases (i.e. xenon or argon). In two studies, magnesium administration did not result in a better survival or neurological outcome for CA patients [114, 115]. However, hypothermia was not used in these studies, while the combination of magnesium with cooling procedures has been shown to result in the highest neuroprotective effects [116]. Importantly, many questions remain unanswered on the optimal timing to initiate magnesium perfusion, optimal dosing and circulating levels and potential side-effects, which may explain the negative results of magnesium therapy after subarachnoid hemorrhage and traumatic brain injury [117, 118]. Noble gases have been proved to reduce the extent of neurological damage after ischemia in animal models of CA [119, 120]. The administration of Xenon was also feasible in the human setting, reporting both no adverse events and an improvement of cardiovascular function after CA [121]. Unfortunately, we still do not have any human data describing the potential neurological benefits of such treatment.

Mitochondrial dysfunction could be attenuated by the administration of erythropoietin (EPO), the principal hematopoietic hormone regulating erythropoiesis, which shows also anti-apoptotic, anti-inflammatory and anti-oxidant properties [122]. In a swine model of VF, high-dose EPO administration during CPR reduced post-resuscitation myocardial dysfunction and improved cardiac function [123]. However, post-ischemic EPO administration in rats exposed to CA exerted no protective effect on hippocampal neurons [124]. One human study has compared the effects of 90.000 UI of EPO given during CPR to an historical matched control group [125]. The EPO group had higher rates of ROSC (92 versus 53 %, p = 0.006) and hospital survival (54 versus 20 %, p = 0.011) when compared to the control group. In another study [126], EPO therapy was associated with a trend towards higher full neurological recovery (55 versus 38 %) when compared to an historical cohort. Unfortunately, a recent unpublished randomized clinical trial (RCT) found no benefits of EPO administration on comatose survivors after CA when compared to placebo (Cariou et al.—presented at the 27th ESICM congress—Barcelona, October 2014).

Reperfusion injury leads to mitochondrial dysfunction also through the opening of a nonspecific pore in the inner mitochondrial membrane, known as the mitochondrial permeability transition pore (MPTP) [127]. This phenomenon causes the loss of ionic homeostasis and ultimately cell swelling and death. The inhibition of the MPTP opening may provide some protection against reperfusion injury; importantly, this may be mediated by a direct interaction of cyclosporine A (CsA) with a protein located on the MPTP, called cyclophilin-D [127]. In a murine model of CA, CsA was effective in reducing myocardial dysfunction when given at the onset of resuscitation but not after ROSC [128]. In one human study conducted in patients suffering from acute myocardial infarction, the administration of a 2.5 mg/kg bolus of CsA before percutaneous coronary intervention was associated with a significant reduction in biomarkers of myocardial injury (i.e. troponin I) and the extent of ischemic areas on cardiac magnetic resonance imaging [129]. Nowadays, no clinical studies have evaluated the effects of CsA on the neurological recovery of CA survivors yet.

Also, mitochondrial dysfunction can be modulated through the nitric oxide (NO)-related pathways; NO inhibits ROS-producing enzymes and directly scavenges ROS production [130]. Other potential beneficial effects are the direct vasodilation of coronary arteries, which could improve cardiac function in this setting. Experimental models have suggested a protective role of intravenous NO-donors or inhaled NO both on cardiac and neurological function after CA [131, 132]. Unfortunately, only one pilot study showed the feasibility and safety of low-dose nitrite infusion in cardiac arrest survivors, but did not report any improvement in outcome [133].

Finally, recent data suggest that the early administration of corticosteroids after in-hospital CA was associated with an increased survival rate and reduced occurrence of extra-cerebral organ failures [134]; whether these effects were secondary to an anti-inflammatory effect or the treatment of a relative adrenal insufficiency [135], it remains to be further evaluated. Similarly, abnormalities of peripheral microcirculation, similar to those found in septic patients, have been described after CA [136]; the pathophysiology of such alterations as well as their role on patients’ outcome and the various therapeutic options to manipulate the microvascular flow have to be better characterized in future studies.

### Neurological assessment of brain damages

#### Minimal consciousness: classification and prognosis in CA patients

##### Steven Laureys and Didier Ledoux

Successful resuscitation after cardiac arrest or cerebral hypoxia can have an extremely wide variety of functional and cognitive consequences, ranging from transient cognitive or motor dysfunction lasting several weeks or months; to awakening after coma in a state of unawareness or inability to communicate lasting months to years or decades (e.g. as seen in vegetative or minimally conscious states) [137]. At present, the medical and economic impacts of differences in clinical management (including therapeutic hypothermia), care pathways and disparate health policies within Europe remain unclear.

**Fig. 8:**
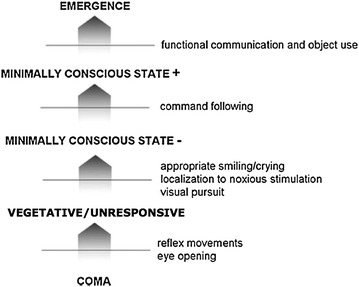
Timeline of events that may occur after anoxic coma

Figure 8 illustrates the timeline of events that may occur after anoxic coma. When cerebral hypoxia leads to a prolonged loss of consciousness (lasting >2 h to differentiate from syncope), the patient is considered to be in coma. Patients in coma will never open the eyes, even if stimulated by a loud noise or intense noxious stimulus, and will only show reflex movements. Coma will not last longer than a couple of days to weeks. Most patients who show good recovery will rapidly show signs of consciousness and functional communication. Some patients, however, will evolve to brain death (i.e., irreversible coma with absent brainstem reflexes) and organ donation should be discussed [138]. Many patients will remain with some brainstem function (i.e., will not be brain death), but clinical and complimentary testing shows that there is no reasonable chance for a meaningful recovery and the decision is made to withhold or withdraw treatment. In these cases, organ donation after cardiac death can be discussed.

Patients who survive their coma may awaken (i.e., open the eyes) without any behavioural sign of consciousness (i.e., only show reflex or automatic movements), a condition coined “persistent vegetative state” or “unresponsive wakefulness syndrome” (PVS/UWS) [139] or without recovery of functional communication or functional object use, a condition referred to as “minimally conscious state” (MCS) [140]. In contrast to coma, such chronic disorders of consciousness can last for many months to years and at present there are no reliable epidemiological data regarding these challenging patients. The heterogeneous MCS group is subcategorized in MCS—when patients only show non-reflexive behaviour such as eye tracking, orientation to pain or contingent behaviour to specific stimuli (e.g. smiling exclusively in the presence of a family member) and MCS+ when a reproducible (albeit often inconsistent) response to command can be observed [141].

When patients remain in PVS/UWS for over 3 months after the cardiac arrest, the condition is considered irreversible and life-sustaining treatment (i.e., artificial hydration and nutrition) may be considered as futile and hence be withdrawn. At present, the chances of recovery after post-anoxic MCS are considered to be better than for PVS/UWS but remain ill defined. Many patients who recover from coma or related disorders of consciousness will show cognitive dysfunction and may remain institutionalized or dependent of others for many activities of daily living, but few reliable data exist regarding their remaining quality of life.

Recent advances in automated clinical EEG analysis [142], combined EEG transcranial magnetic stimulation studies [143] and structural and functional neuroimaging have permitted to better document the clinical diagnosis [144] and levels of consciousness in patients with severe post-anoxic encephalopathy [145]. Several studies also show their value in predicting the chances of recovery after cardiac arrest (e.g., the assessment of EEG reactivity [142]; the quantification of white matter damage using MRI diffusion tensor imaging [146] or PET imaging of residual cortical metabolism [147].

At present, there are no evidence-based guidelines regarding the treatment of patients with chronic anoxic disorders of consciousness [148]. In terms of noninvasive intervention, transcranial direct current stimulation has recently been shown to be of potential interest [149]. A better understanding of the temporal dynamics of possible residual neural plasticity following anoxic coma and related conditions will permit to improve their clinical management (including treatment for possible pain perception [150]) and to rationalize our medical interventions [151] acute and chronic care pathways and end-of-life decisions [152].

#### Clinical tools

##### Mauro Oddo

The ideal tool for coma prognostication is the one yielding the lowest false-positive rate (FPR) for poor outcome. The definition of FPR is 1—specificity, whereby the “perfect” predictor would allow 100 % (i.e. 1.0) specificity and an FPR for poor outcome of 0 and limit as far as possible the risk of false predictions. Clinical examination comprises tools for the assessment of neurological responses/reflexes, including the Glasgow Coma Scale (GCS), brainstem reflexes and the comprehensive Full Outline of Unresponsiveness (FOUR) score. Clinical examination is an essential step for coma prognostication after CA [153]. However, clinical examination may be misleading, partly because of the effect of mild induced hypothermia and sedation on neurological responses. In particular, using the motor component of the GCS, it was found that motor response to pain may be delayed up to 5–6 days following CA [154]. Motor reaction to pain no better than extension indeed yields an unacceptably high FPR (10–20 %) for poor prognosis [155]. Apart from GCS, the assessment of brainstem reflexes (pupillary and corneal reflexes in particular) is crucial. Corneal and pupillary reflexes have very high specificity: bilaterally absent pupillary/corneal reflexes yield an FPR <1–2 % for poor prognosis, while their FPR for good prognosis is relatively high (40–50 %) [155].

The predictive value of pupillary reactivity is underlined by recent systematic meta-analyses [155, 156]. In this setting, newly available automated infrared pupillometers hold great promise, because they allow quantitative measurement of the pupillary response. One single-center study recently showed quantitative pupillometry is superior to standard pupillary examination for post-CA coma prognostication and performs comparably to electro-physiological exams (including EEG and SSEP) [157].

In summary, careful and complete neurological examination is the first and still valid tool for coma prognostication [158]. Clinical tools should be integrated into a multimodal approach, which ideally also include electrophysiology (EEG reactivity and/or SSEP) and blood biomarkers (e.g. NSE). Using this multimodal approach, prediction of 3-month survival and neurological outcome might approach 90 % accuracy [159]. Application of such approach is supported by several recent independent studies and can be recommended in ICU practice [160].

#### New electrophysiological tools: continuous and simplified electroencephalography (aEEG)

##### Hans Friberg

Electroencephalography (EEG) is a commonly used method for identification of seizures and for estimation of prognosis in comatose survivors after cardiac arrest [161]. Limitations with an EEG-investigation include its sensitivity to sedatives and the fact that a single conventional EEG depicts the actual status during a limited time, commonly 20–30 min. Another problem has been the lack of consensus regarding EEG terminology and the absence of a uniform classification system, a problem that may be resolved by implementation of a standardized critical care EEG-terminology as recently proposed [162].

EEG-patterns that are strongly associated with a poor outcome after cardiac arrest and considered “malignant patterns” include a generalized suppression to <20 µV, burst-suppression pattern with generalized epileptiform activity, or generalized periodic complexes on a flat background [163]. In addition, a non-reactive EEG background pattern after rewarming from hypothermia treatment has been found to be a predictor of poor outcome [164]. A reactive EEG-pattern on the other hand, and a return of a continuous EEG background pattern after cardiac arrest are both strongly associated with recovery and a good outcome [163, 164].

Continuous EEG (cEEG) provides dynamic information and can be used to monitor evolution of EEG-patterns and to detect seizures in the postischemic brain [165]. A traditional multichannel montage is commonly used in adult ICUs, while a simplified EEG-montage with few channels and trend analysis is more common in neonatal ICUs [166]. In order to reach general use, cEEG needs to be simple, possible to apply bedside and cost-effective.

**Fig. 9:**
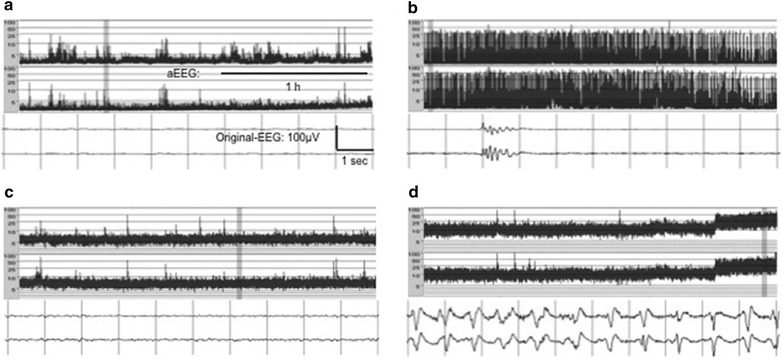
A simplified electroencephalogram (EEG) with two original EEG curves (*lower panel*), in combination with an amplitude integrated EEG (aEEG) trend curve (*upper panel*). Four dominating EEG patterns after cardiac arrest are shown: **a** flat, **b** suppression-burst, **c** continuous and **d** electrographic status epilepticus. From Friberg et al. [161]. Reprinted with permission

We applied aEEG in consecutive cardiac arrest patients [167]. The aEEG curve facilitates rapid surveillance of extended time periods, but interpretation of patterns is done on the original EEG-tracings, one from each hemisphere. The relative simplicity of cEEG with a reduced montage and aEEG trend analysis makes it attractive to generalists in the ICU. It is applied bedside by the ICU-staff (registered nurse) and data are linked to the neurophysiologists, who will assess the collected data on a regular basis and on demand. In order to facilitate for clinicians, four dominating EEG-patterns after cardiac arrest were identified and described (Fig. 9) [167]. Our experience is that cEEG with a simplified montage and aEEG trend analysis is well suited to follow transitions in background patterns and has acceptable sensitivity to detect clinically relevant electrographic seizures. A return of a continuous EEG-pattern at normothermia is an early and strong prognostic indicator with a positive-predictive value for a good outcome of 0.87 (95 % CI 0.76–0.94) while other patterns at normothermia indicate a poor prognosis with a negative predictive value of 0.91 (95 % CI 0.76–0.98) [167]. The clinical benefit of treating electrographic seizure activity after cardiac arrest is yet to be proven, but simplified cEEG is a reasonable routine method to diagnose seizure activity and to guide treatment.

In conclusion, simplified continuous EEG (cEEG) with trend analysis (aEEG) is a feasible and probably cost-effective method to improve care after cardiac arrest [168]. Collaboration with neurologists and neurophysiologists and education of staff are keys to success. We foresee that simplified cEEG will be part of routine bedside monitoring of comatose survivors after cardiac arrest in the near future.

#### Neurological assessment in the long-term follow-up after cardiac arrest

##### Stephane Legriel

Half the survivors after CA have long-term follow-up cognitive impairment affecting preferentially memory and executive functioning, anxiety, depression, and finally quality of life. Surprisingly, there is no prospective study with extensive neuropsychological assessment and longitudinal follow-up allowing to confirm these retrospective data [169]. Moreover, the era of therapeutic hypothermia may have changed not only long-term outcomes, but also its early prognosis assessment [170]. Thus, three important questions can be raised.

*What tools do we use to describe the long*-*term outcome and are these tools sensitive?* Whereas a recent review demonstrated a broad heterogeneity in the outcome measures utilized in clinical trials, the Cerebral Performance Category (CPC) score is the recommended measurement of long-term outcome after CA [170]. The question of reliability of the CPC score has been evaluated in several studies. There was a strong association between the CPC score at hospital discharge and long-term outcome. Moreover, the CPC score demonstrated good correlation with various neuropsychological evaluations in extensive cognitive battery tests, memory tests, adaptative behavior and quality-of-life evaluations. Thus the CPC could be considered as a gross indicator of long-term functional outcome after CA.

**Fig. 10:**
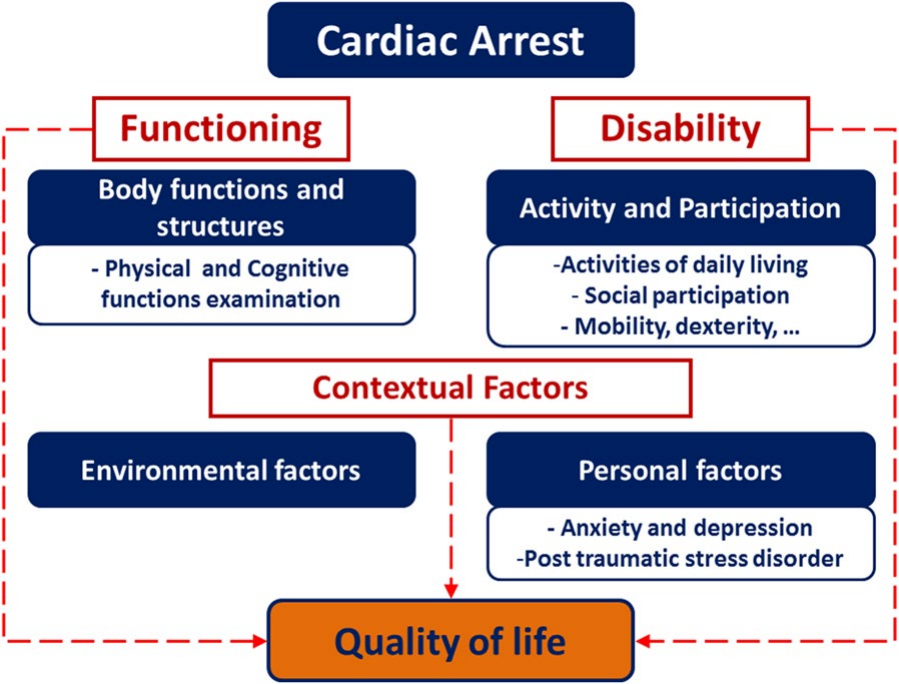
Assessment of health condition in the long-term follow-up after cardiac arrest (adapted from World Health Organization [171])

*How could we evaluate the outcome during the long*-*term follow up?* According to the World Health Organization guidelines, evaluation of health after CA should associate body functions and structures, with physical examination and cognitive functions, activity and participation, personal factors such as anxiety and depression, and so quality of life (Fig. 10) [171, 172]. The American Academy of Clinical Neuropsychology recently recommended evaluation of the following items during a neuropsychological evaluation: intellectual functioning, attention and executive functioning, memory and learning, language and communication, visual–spatial cognition and visual–motor praxis, motor and sensory function, mood, conduct, personality, quality of life, adaptative behavior (activities of daily living), social–emotional awareness and responsivity, psychopathology (psychotic thinking or somatization), motivation and effort [173]. Thereby, a formal neuropsychological evaluation covers the whole spectrum of objectives of health evaluation.

*What approach should we use to make an early prognosis of long*-*term outcome?* All the parameters provided in 2006 by the American Academy of Neurology for prediction of outcome in comatose survivors after cardiopulmonary resuscitation have been challenged since the application of therapeutic hypothermia. Predictors based on clinical examination within 72 h after CA have demonstrated higher false-positive rates [164]. Exceptional survivors have been described despite bilateral absence of N20 waves in median nerve somatosensory evoked potentials. Neuron Specific Enolase demonstrated variable cut-off values associated with poor outcome ranging from 25 to 49.5 ng/mL on day 2 after cardiac arrest, with one survivor with an NSE value of 110 ng/mL. On day 3, variability was greater ranging from 10.6 to 97 ng/mL. Protein S100B was also associated with variability on day 1 after CA [156]. Postanoxic status epilepticus has been demonstrated as an independent factor associated with poor outcome in retrospective and prospective studies [174, 175], but survivors with good long-term functional outcome have been described in 6 % of cases [175]. Continuous background pattern and reactivity during TH or normothermia has been associated with recovery and EEG plays now an increasingly important role in prognostication of coma after CA. Thus, continuous EEG has been suggested to assist with prognostication of coma after CA [176]. Finally, literature data clearly favour a multimodal approach of prognostication after CA.

In conclusion, there is a broad variability in tools used for evaluation during the long-term follow and a lack of standardization. The cerebral performance category scale is an efficient but imperfect surrogate tool to describe long-term outcome after CA. The neuropsychological evaluation seems able to cover the whole spectrum of objectives of health evaluation needed to evaluate the long-term outcome. Early prognosis of long-term outcome at the era of therapeutic hypothermia should be based on a multimodal approach in order to minimize false predictions.

### Public healthcare

#### Stopping cares leads to organ donation?

##### Philippe Hantson

There is still currently a shortage of organs for transplantation and accordingly, different strategies have been developed, including the possibility of organ donation after cardiocirculatory death (DCD) as opposed to donation after brain death (DBD). Following the Maastricht workshop on non-heart beating donation in 1995, several categories of donors have been identified, but the acceptance of such procedures still varies largely among the different European countries. There is no doubt that the ratio DCD/DBD will continue to increase over time.

The ICUs are mainly concerned by categories II (unsuccessful resuscitation) and III (awaiting cardiac arrest). Category III is a controllable situation that could be managed either in the ICU or in the operating room. The possibility of organ donation under category III is also linked to ethical discussions regarding the management of the “end of life”, with a clear distinction between the concepts of euthanasia and withdrawal/withholding of some specific cares [177].

In contrast to DBD where specific protocols are now well accepted, there is a great variability for the DCD protocols regarding the criteria for the determination of death, but also for the definition of the “no-touch” period. This latter corresponds to the time period between determination of death and initiation of organ procurement, and should be theoretically kept as short as possible in order to prevent warm ischemia.

The main ethical difficulties are related to the perceived possibility of conflict of interest for the ICU physician who in one hand could take the decision to withdraw or withhold some specific care, and in a next step could also be authorized to take the decision of organ donation in the same patient. The separation (in time and space) between the two processes is absolutely mandatory. There is a need of full confidence between all the members of the ICU staff taking care of the patient, so that each individual could be convinced that the treatment-withdrawal decision is always made independently from any considerations about the potential of subsequent organ donation.

Another difficult ethical issue is the dying process itself. It remains difficult to predict the time to death by circulatory arrest after the withdrawal of the life-sustaining treatments. When organ donation is not considered, the patient is dying in the ICU, while in case of DCD, death will occur in the operating room, with ideally a short period of hypotension in order to prevent organ damages. But in fact, the dying process has already started several hours or days before the final decision in the ICU. The vision that the DCD procedure could hasten the patient’s death is not totally true, but there are still different perceptions among the ICU physicians. Some of them feel that when comfort therapy with potent sedatives and analgesics (“palliative sedation”) is started in the ICU, the priority is to give a lot of time to the family to accompany the dying person. Other ICU physicians or even scientific societies consider that shortening the dying process with use of medications may sometimes be appropriate, even in the absence of discomfort, and can actually improve the quality of dying. Most of the families express the opinion that the priority should be that the patient would die peacefully, the duration of the dying process being of secondary importance. This is a key issue in the debate “taking care of the patient or taking care of the organs?” [178].

Finally, the wishes expressed by the patient (anticipatively) or by the relatives have clearly to be taken into account. The treatment-withdrawal modalities will clearly be also be influenced by these opinions. When the decision is made to stop the cares in the operating room, it is with the objective that cardiocirculatory death would occur in a delay shorter than one hour and that the procedure of donation would be successful.

In conclusion, organ procurement under Maastricht III category will remain a sensitive issue as no protocol is widely accepted. Each centre has the responsibility to initiate the debate between all the participants involved in critical care and organ transplantation. The main perceived risk is the abolition of the frontier between the cares orientated to the patient and the cares given to the organs.

## Abbreviations

aEEG: continuous and simplified EEG-montage with two channels in combination with amplitude integrated trend analysis
AED: automated external defibrillators
ALS: advanced life support
BLS: basic life support
CA: cardiac arrest
CCPR: conventional CPR
cEEG: continuous electroencephalography
CPR: cardio-pulmonary resuscitation
DBD: donation after brain death
DCD: donation after cardiocirculatory death
DNAR: do-not-attempt-resuscitation
ECPR: extracorporeal CPR
EMS: emergency medical services
ETI: endotracheal intubation
FOUR: full outline of unresponsiveness
GCS: Glasgow coma score
ICU: intensive care unit
IHCA: in-hospital cardiac arrest
MCS: minimally conscious state
MET: medical emergency team
MTH: mild therapeutic hypothermia
NIRS: near-infrared spectrophotometry
OHCA: out-hospital-cardiac arrest
PCI: percutaneous coronary intervention
PE: pulmonary embolism
PEA: pulseless electrical activity
PVS: persistent vegetative state
ROSC: return of spontaneous circulation
RRS: rapid response systems
TH: therapeutic hypothermia
TTM: targeted temperature management
UWS: unresponsive wakefulness syndrome
VF: ventricular fibrillation
WLST: withdrawal of life-sustaining therapy
